# Effect of Poly(styrene-*ran*-methyl acrylate) Inclusion on the Compatibility of Polylactide/Polystyrene-*b*-Polybutadiene-*b*-Polystyrene Blends Characterized by Morphological, Thermal, Rheological, and Mechanical Measurements

**DOI:** 10.3390/polym11050846

**Published:** 2019-05-10

**Authors:** Bocheng Wang, Zheng Tu, Chonggang Wu, Tao Hu, Xiaotao Wang, Shijun Long, Xinghou Gong

**Affiliations:** Hubei Provincial Key Laboratory of Green Materials for Light Industry, Collaborative Innovation Center of Green Light-weight Materials and Processing, and School of Materials and Chemical Engineering, Hubei University of Technology, Wuhan 430068, China; bochengwang@foxmail.com (B.W.); tuzheng666@foxmail.com (Z.T.); cgwu@mail.hbut.edu.cn (C.W.); hutao@mail.hbut.edu.cn (T.H.); xiaotaowang@aliyun.cn (X.W.); longshijun.hp@163.com (S.L.)

**Keywords:** polylactide, polystyrene-*b*-polybutadiene-*b*-polystyrene, compatibilization, poly(styrene-*ran*-methyl acrylate), characterization

## Abstract

A poly(styrene-*ran*-methyl acrylate) (S-MA) (75/25 mol/mol), synthesized by surfactant-free emulsion copolymerization, was used as a compatibilizer for polystyrene-*b*-polybutadiene-*b*-polystyrene (SBS)-toughened polylactide (PLA) blends. Upon compatibilization, the blends exhibited a refined dispersed-phase morphology, a decreased crystallinity with an increase in their amorphous interphase, improved thermal stability possibly from the thicker, stronger interfaces insusceptible to thermal energy, a convergence of the maximum decomposition-rate temperatures, enhanced magnitude of complex viscosity, dynamic storage and loss moduli, a reduced ramification degree in the high-frequency terminal region of the Han plot, and an increased semicircle radius in the Cole–Cole plot due to the prolonged chain segmental relaxation times from increases in the thickness and chain entanglement degree of the interphase. When increasing the S-MA content from 0 to 3.0 wt %, the tensile properties of the blends improved considerably until 1.0 wt %, above which they then increased insignificantly, whereas the impact strength was maximized at an optimum S-MA content of ~1.0 wt %, hypothetically due to balanced effects of the medium-size SBS particles on the stabilization of preexisting crazes and the initiation of new crazes in the PLA matrix. These observations confirm that S-MA, a random copolymer first synthesized in our laboratory, acted as an effective compatibilizer for the PLA/SBS blends.

## 1. Introduction

Polylactide (PLA) has been extensively used in biomedical applications and plastics industry because it has many golden properties, such as degradability, biocompatibility, renewability, good mechanical properties [1,2,3]. However, the low impact strength of PLA prevents its further engineering applications in automotive, electronic, construction fields. Blending with other polymers, such as polybutadiene (PBD) [4], polystyrene-*b*-polybutadiene-*b*-polystyrene (SBS) [5,6], poly(ethylene-*ran*-octane) (POE) [7], poly(butylene adipate-*ran*-terephthalate) (PBAT) [8], acrylonitrile-butadiene-styrene terpolymer [9], polyurethane [10], poly(vinyl alcohol) [11], ethylene-vinyl acetate copolymer [12], poly(butylene succinate) (PBS) [13,14], high-density polyethylene (HDPE) [15], natural rubber [16], poly(ethylene oxide) [17], high-impact polystyrene (HIPS) [18], hydrogenated SBS [19], is an effective and economical approach to the improvement of the impact strength of PLA. Since most of the above polymers are immiscible with PLA, their toughening effects are largely restricted. A number of methods have been used to enhance the compatibility of the PLA/polymer blends. For instance, some researchers have used chemical methods to modify the polymer toughening agents of PLA, thus producing epoxidized PBD [4], glycidyl methacrylate (GMA)-grafted POE [7], PLA-*b*-PBS-*b*-PLA [14], PLA-*b*-polyethylene-*b*-PLA [20], etc. The chemical method is not a cost-effective strategy because all toughening component need to be modified. If a small quantity of a third component is used as a compatibilizer to improve the compatibility of PLA blends, the cost would be decreased significantly. Therefore, most of the researchers have used a copolymer as a compatibilizer to modify the blends. For example, Ding et al. [8] used PLA-poly(ethylene glycol)-PLA triblock copolymer as a compatibilizer to modify PLA/PBAT blends. Supthanyakul et al. [13] used poly(butylene succinate-*ran*-lactic acid) to act as a compatibilizer of PLA/PBS blends and found a significant decrease in the *T*_g_ of the PLA matrix as well as an increase in the elongation at break of the blends. Gallego et al. [15] used poly(lactide-*ran*-carboxyl-and-hydroxyl-containing polyethylene) (LA-CHPE) copolymer and PLA-*g*-polyethylene (PE) graft copolymer, respectively, to modify PLA/HDPE blends and found that the compatibilization efficiency of the LA-CHPE synthesized by ring-opening polymerization was higher than that of PLA-*g*-PE. Sikka et al. [21] used poly(styrene-*ran*-methyl methacrylate) copolymers (S-MMAs) to modify a polystyrene (PS)/poly(methyl methacrylate) (PMMA) laminate interface; they found that the PS/PMMA modified with the S-MMA (0.73/0.27 mol/mol) showed denser oblique crazes than that modified with the S-MMA (0.48/0.52 mol/mol), resulting in a higher fracture toughness.

In order to improve the compatibility of the PS- and HIPS-reinforced PLA blends, Gong et al. in our group synthesized for the first time a poly(styrene-*ran*-methyl acrylate) (S-MA) and successfully used it as a compatibilizer to significantly improve the mechanical properties of PLA/PS [22] and PLA/HIPS [18] blends. Specifically, the maximum mechanical properties of PLA/PS occurred at an S-MA content of 0.5 wt %; upon incorporation of 1.0 wt % of the S-MA, the tensile and impact strengths of the PLA/HIPS were improved by 95.3% and 104.8%, respectively. SBS is a thermoplastic elastomer with excellent tensile properties and good processability, which has been used to toughen HIPS [23], polypropylene (PP) [24], poly(butylene terephthalate) [25], PS [26], etc. In recent years, PLA/SBS blends have received more attention due primarily to the renewability and biodegradability of the PLA matrix. For instance, Wu et al. [6] investigated the effects of SBS inclusion on the crystallization rate, elongation at break, and impact strength of PLA. Four kinds of rubbery toughening components, including SBS, ground tire rubber, POE, and GMA-grafted POE, were blended with PLA at a mass ratio of 15/85; the PLA/SBS blend displayed the highest impact strength as well as elongation at break [27]. Nevertheless, PLA/SBS blends are immiscible. To compatibilize PLA with SBS, Wang et al. [5] prepared epoxidized SBSs at different degrees by a chemical reaction, in which the reaction required a long period of time (15 h) and the products needed a series of post-processing; moreover, a large amount of toluene was consumed during the reaction as the solvent. Obviously, this method is costly, unsuitable for mass production, and not eco-friendly.

Since Eguiburu et al. [28] reported that PLA/poly(methyl acrylate) (PMA) was apparently a miscible blend, an S-MA was used in this work to compatibilize SBS-toughened PLA blends. S-MA was already used by our group for the compatibilization of PLA/PS [22] and PLA/HIPS blends [18]. The synthesis of S-MA was carried out in water phase by surfactant-free emulsion copolymerization, which is facile and whose product is relatively of high purity because of the absence of surfactant and demulsifier. To obtain highly toughened PLAs, 5 or 10 wt % of SBS was blended with PLA, with different loading levels of S-MA as a compatibilizer. To probe the compatibility of the compatibilized PLA/SBS blends, their phase morphology, thermal, rheological, and mechanical properties were investigated and compared with those of the uncompatibilized blends.

## 2. Materials and Methods 

### 2.1. Materials

Styrene (≥99.5%), methyl acrylate (≥98.5%), acetone (≥99.5%), potassium persulfate (≥99.5%), and ethanol (≥99.7%), all of analytical grade, were purchased from Sinopharm Chemical Reagents Co., Ltd., China. PLA (3052D), primarily poly(_L_-lactide) (PLLA) with 4 mol % of _D_-lactic acid units, an ${\bar{M}}_{w}$ of 190,000, and a polydispersity index of 1.7 [29,30], was supplied by NatureWorks LLC, USA, with a melt flow rate of 14 g/10 min (210 °C, 2.16 kg) (ASTM D1238) and a specific gravity of 1.24 (ASTM D792). A linear SBS (YH-791), with 30 wt % of PS and 70 wt % of PBD and an ${\bar{M}}_{w}$ of 160,000 [31], was obtained from Baling Petrochemical Co., SINOPEC Group, Yueyang, China. Distilled water was home-made in our laboratory using a stainless-steel water distiller. The styrene was distilled under reduced pressure to remove any possible polymerization inhibitor(s) and impurities. Potassium persulfate was purified by recrystallization in a 0 °C ice-water bath from 50 °C distilled water. All the other chemicals were used as received without any further purification.

### 2.2. Synthesis of S-MA by Surfactant-Free Emulsion Copolymerization

A surfactant-free emulsion free-radical copolymerization was conducted to synthesize the S-MA compatibilizer, with a nominal S/MA mole ratio of 75/25, an ${\bar{M}}_{w}$ of 259,000, and a polydispersity index of 3.6, according to the procedure described in references [18,22].

### 2.3. Preparation of S-MA-Compatibilized PLA/SBS Blends

Prior to melt blending, PLA and SBS were dried at 80 °C under vacuum for at least 12 h. Two PLA/SBS mass ratios, 95/5 and 90/10, were used with S-MA compatibilizer contents of 0 (uncompatibilized), 0.5, 1.0, 2.0, and 3.0 wt % relative to the total mass of PLA and SBS. Melt blending of PLA, SBS, and S-MA was carried out in a co-rotating twin-screw extruder (Harbin Hapro Electric Technology Co., Ltd., China, R200C) at a screw speed of 50 rpm with a temperature profile of 160/180/190 °C from hopper to die. The extrudates were then quenched in a cold-water bath, subsequently pelletized using a grinder, and finally dried in vacuo at 80 °C for at least 12 h.

To compare similar preparation histories, neat PLA was extruded following the same procedure as described above for the PLA/SBS/S-MA blends.

### 2.4. Field-Emission Scanning Electron Microscopy (FE-SEM)

The fractured surface topography of the impact fractured specimens was observed under different magnifications using a field-emission scanning electron microscope (Hitachi, S4800, Tokyo, Japan) at an acceleration voltage of 10 kV. Prior to SEM observation, all the fractured surfaces of the specimens were coated with gold using a sputter coater (Quorum, K575X).

### 2.5. Differential Scanning Calorimetry (DSC)

The non-isothermal crystallization behavior of the blends and neat PLA was investigated using a differential scanning calorimeter (TA Instruments, Q2000, New Castle, DE, USA). DSC scans were conducted under N_2_ atmosphere with heating and cooling rates of 20 °C/min. Small amounts (~5.0 mg) of pellet samples were encapsulated by a sample maker into an aluminum pan with an aluminum lid. The samples, placed in the DSC cell, were heated to 200 °C, then held there for 3 min to eliminate any possible preparation history, subsequently cooled to 20 °C, and finally heated again to 200 °C. The glass transition temperature (*T*_g_), “cold” crystallization (i.e., recrystallization) temperature (*T*_cc_), and melting temperature (*T*_m_) were analyzed in the DSC trace from the second heating cycle. The relative degree of crystallinity (*X*_c_) of the PLA matrix was obtained from its melting enthalpy (Δ*H*_m_) using the following equation,

(1) $$X_{c}\;=\;\frac{{\Delta H}_{m}}{{\Delta H}_{m}^{0}\varphi_{\mathrm{PLA}}}\;\times \;100\%$$

where ${\Delta H}_{m}^{0}$ is the melting enthalpy of 100% crystalline PLA (93.0 J/g [32]), and *φ*_PLA_ is the mass fraction of PLA in the blends.

### 2.6. Thermogravimetric Analysis (TGA)

The thermal decomposition behavior of the blends, neat PLA and SBS, was studied with a thermogravimetric analyzer (TA Instruments, Q600). A small amount (~5.0 mg) of each material was heated from 30 to 600 °C at a rate of 10 °C/min under N_2_ atmosphere.

### 2.7. Rheological Measurements

The rheological properties of the blends, as well as those of neat PLA and SBS, were investigated in the oscillatory shear mode using a rotational rheometer (TA Instruments, DHR-2) with a 25 mm parallel-plate fixture and a 1.8 mm gap between the plates. A small strain amplitude of 0.1% was employed to ensure a linear viscoelastic behavior of the specimens. Before the test, pellets of each material were compression-molded at 180 °C and 10 MPa into discoid specimens of 25 mm in diameter and 2 mm in thickness. Frequency-sweep tests were performed on the specimens from 10^−2^ to 10^2^ Hz at 180 °C under N_2_ atmosphere.

### 2.8. Mechanical Properties Measurements

An injection molding machine (Shanghai Xinshuo Precision Machinery Co., Ltd., China, MiniJet WZS10D) was used to mold standard tensile and Charpy notched impact specimens, with barrel and mold temperatures of 180 and 20 °C, respectively. The tensile properties of the prepared blends and neat PLA were measured with a universal testing machine (MTS Industrial Systems (China) Co., Ltd, CMT-4202) according to the ISO 527-1 testing standard, with a gauge length of 25 mm and a crosshead speed of 5 mm/min. In each of the stress–strain curves, the Young’s modulus was read as the slope of the tangent at the origin, and the yield strength was defined as the tensile strength, except that, for neat PLA not exhibiting a typical stress–strain yield behavior, the break strength was defined as the tensile strength. For each composition of material, arithmetic means, in Young’s modulus, tensile strength, and elongation at break, from at least five measurements, as well as the typical stress–strain curve having a tensile strength most approximate to the mean one, were taken as the data for analysis. 

The impact strengths of the specimens were tested using a Charpy notched impact tester (Chengde Material Testers Plant, Chengde, China, XCJ-4) according to the ISO 179-1 testing standard. The notched impact-strength values (kJ/m^2^), *a*_cN_, were evaluated from the corrected energy (J), *E*_c_, by use of the following equation

(2) $$a_{\mathrm{cN}}\;=\;\frac{E_{c}}{{hb}_{N}}{\;\times \;10}^{3}$$

where *h* is the thickness (mm) of the impact specimens, and *b*_N_ the remaining width (mm) in the breadth direction of the notched impact specimens. For each composition of material, an arithmetic mean of impact strength (kJ/m^2^) from at least five measurements was taken for data analysis.

## 3. Results and Discussion

### 3.1. Effect of S-MA Compatibilization on the Morphology of PLA/SBS Blends

**Figure 1:**
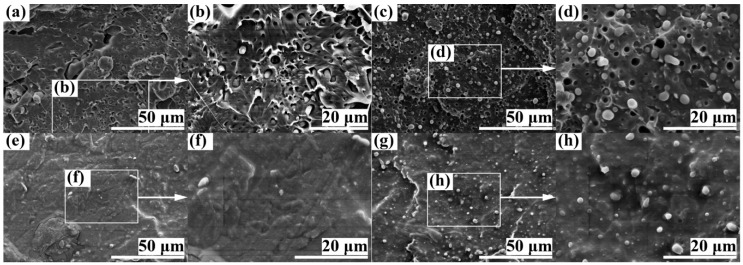
Scanning electron microscopy (SEM) micrographs of the impact-fractured surfaces of polylactide (PLA)/polystyrene-*b*-polybutadiene-*b*-polystyrene (SBS) (95/5 w/w) blends (**a**–**d**) uncompatibilized and (**e**–**h**) compatibilized with 1.0 wt % poly(styrene-*ran*-methyl acrylate) (S-MA). Images (**a**), (**b**), (**e**), and (**f**) represent the surface topography in the “skin” of a typical injection-molded Charpy notched impact specimen; images (**b**) and (**f**) are magnifications of images (**a**) and (**e**), respectively; images (**c**), (**d**), (**g**), and (**h**) show the surface topography in the “core” of the same specimen; images (**d**) and (**h**) are magnifications of images (**c**) and (**g**), respectively.

Figure 1 presents the fractured surface topography of a typical impact-fractured specimen of PLA/SBS (95/5 w/w) compatibilized with 1.0 wt % of S-MA (Figure 1e–h) compared with that of uncompatibilized PLA/SBS (95/5 w/w) (Figure 1a–d). The SBS dispersed phases appeared in the images as dark holes and bright spheroids, especially in the magnified images b, d, f, and h of images a, c, e, and g respectively, which was probably due to the “pull-out from the PLA matrix” and “break within the SBS phase itself” effects during the impact fracture of the specimens. It is worth noting that the fractured surface topography (images a and b or e and f) in the “skin” of the injection-molded impact specimens was quite different from that (images c and d or g and h) in the “core”, in that, likely owing to a strong shear orientation effect in the “skin”, the former (especially image b) exhibited ellipsoidal, distorted ellipsoidal, and even irregular SBS dispersed-phase particles compared with primarily spherical SBS particles in the latter.

Interestingly, in the “skin” (Figure 1a,b,e,f) the SBS dispersed-phase particles were almost indiscernible in 1.0 wt % S-MA-compatibilized PLA/SBS (95/5 w/w) blend (Figure 1f), while there were distinct elongated particles in the uncompatibilized counterpart (Figure 1b); this contrast indicates an effective compatibilization of the blend by S-MA that resulted in a more uniform and refined two-phase morphology in Figure 1f than in Figure 1b. Likewise in the “core” (Figure 1c,d,g,h), a similar yet more quantitative observation (<1.0 μm in Figure 1h vs ~2.0 μm in Figure 1d) was made compared with Figure 1f vs b in terms of the mean diameter of the SBS dispersed phase; more importantly, the compatibilized blend (Figure 1h) contained essentially bright spheroids from the “break” effect, whereas the uncompatibilized counterpart (Figure 1d) included not only bright spheroids but also dark holes largely from the “pull-out” effect. These verify that the incorporated 1.0 wt % of S-MA did act as an effective compatibilizer for the PLA/SBS (95/5 w/w) blend.

**Figure 2:**
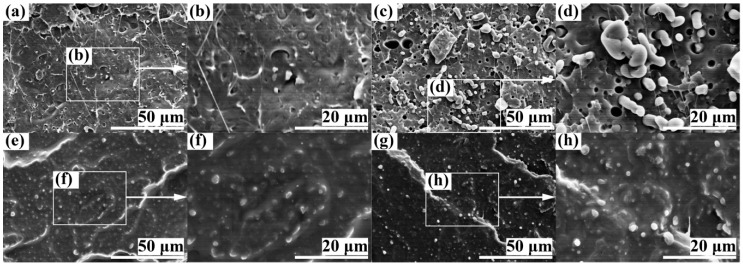
SEM micrographs of the impact-fractured surfaces of PLA/ SBS (90/10 w/w) blends (**a**–**d**) uncompatibilized and (**e**–**h**) compatibilized with 1.0 wt % of S-MA. Images (**a**), (**b**), (**e**), and (**f**) represent the surface topography in the “skin” of a typical injection-molded Charpy notched impact specimen; images (**b**) and (**f**) are magnifications of images (**a**) and (**e**), respectively; images (**c**), (**d**), (**g**), and (**h**) show the surface topography in the “core” of the same specimen; images (**d**) and (**h**) are magnifications of images (**c**) and (**g**), respectively.

Figure 2 demonstrates the fractured surface topography of PLA/SBS (90/10 w/w) compatibilized with 1.0 wt % of S-MA (Figure 2e–h) as opposed to the uncompatibilized PLA/SBS (90/10 w/w) (Figure 2a–d); in it, images a and e as well as their magnifications (images b and f, respectively) show the “skin” while the others (images c and g, as well as their magnifications, images d and h, respectively) were obtained from the “core”. Generally, observations similar to those from Figure 1 were made from Figure 2. Nevertheless, there was a gradual transformation of SBS dispersed-phase morphology (basically, its shape) from spheroids to rods with an increase in the SBS content from 5 wt % (Figure 1) to 10 wt % (Figure 2); moreover, the size of the SBS phase apparently became larger in Figure 2. For instance, compared with Figure 1d, more rod-like SBS particles were observed in Figure 2d with a larger size; also, Figure 2f displays a more pronounced (i.e., coarsened) SBS dispersed phase than Figure 1f.

### 3.2. Effect of S-MA Compatibilization on the Thermal Transition Behavior of PLA/SBS Blends

**Figure 3:**
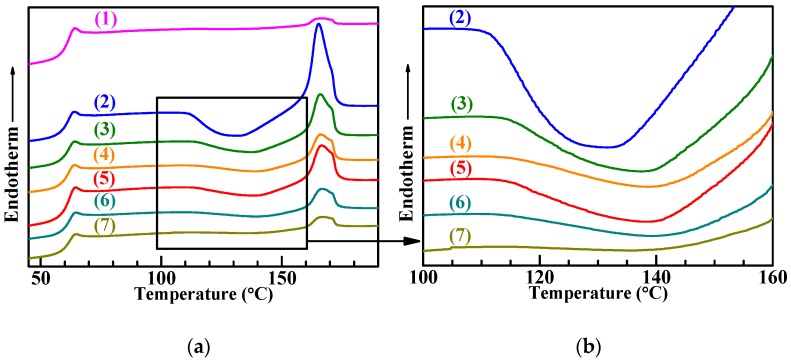
(**a**) Differential scanning calorimetry (DSC) thermograms and (**b**) magnification of the squared region in (a) in the second heating cycle at a rate of 20 °C/min for (1) (neat) PLA, (2) a blend of PLA and a SBS (95/5 w/w), PLA/SBS (95/5 w/w) blend compatibilized with (3) 1.0 wt % and (4) 3.0 wt % of S-MA, (5) a blend of PLA and SBS (90/10 w/w), PLA/SBS (90/10 w/w) blend compatibilized with (6) 1.0 wt % and (7) 3.0 wt % of S-MA.

Figure 3 shows the DSC curves in the second heating cycle for (neat) PLA and the PLA/SBS blends with different compositions (95/5 and 90/10 w/w), compatibilized with different amounts (0, 1.0, and 3.0 wt %) of S-MA (Figure 3a) and a magnification of the squared region in Figure 3a (Figure 3b), whose thermal transition behaviors were analyzed as summarized in Table 1. It is seen from the table that the *T*_g_ of the PLA matrices of all materials fell invariably between 61.4 and 62.0 °C, possibly due to the fact that the small mass fractions of the added SBS (5 and 10 wt %) and S-MA (0, 1.0, and 3.0 wt %) had no significant effect on the *T*_g_ of the respective PLA matrices. A small endothermic peak was found in Figure 3a at the end of the glass transition step of each material, since the residual strain formed from the relatively high cooling rate (20 °C/min) led to enthalpy recovery during the subsequent heating process [33]. It was observed that there was no discernible melt crystallization transition during the first cooling cycle (not shown here), which dictates that the “cold” crystallization enthalpy (Δ*H*_cc_) was comparable to the melting enthalpy, Δ*H*_m_, for the PLA matrices of all materials, as seen from Table 1. Under this circumstance, the relative degrees of crystallinity, *X*_c_, evaluated by Equation (1) from Δ*H*_m_, were reflected exclusively by the “cold” crystallinity.

As shown in Table 1, upon incorporation of 5 wt % SBS, the “cold” crystallization temperature, *T*_cc_, of the PLA matrix was reduced from 137.2 to 132.1 °C, indicating an enhancement of *X*_c_ primarily due to an acceleration of the crystal growth presumably from the plasticization effect of the flexible SBS chains. Further for the PLA/SBS (95/5 w/w) blends, with steady increases in S-MA compatibilizer content from 0 to 3.0 wt %, the *T*_cc_ of the PLA matrices started to rise monotonously from 132.1 to 139.2 °C (Table 1), revealing monotonic decreases in *X*_c_ essentially as a result of the so-called “thinning” effect [6], i.e., increases in the volume fraction of the amorphous PLA–SBS interphase from the gradually refined two-phase morphology of the increasingly compatibilized PLA/SBS blends. More quantitatively, *X*_c_, given in Table 1, of the PLA matrix was enhanced dramatically from 1.4% to 19.5% upon addition of 5 wt % of SBS, that is, the melt crystallinity of the neat PLA was very weak (*X*_c_ < 2%) owing to its low chain tacticity, the steric-hindrance effect of its methyls, the dipole–dipole interactions between its chain segments, and its relatively high melt viscosity [34], while that of the PLA/SBS (95/5 w/w) blend was much stronger (*X*_c_ ~ 20%). This sharp contrast, as explained for the *T*_cc_ change upon SBS addition, again, was probably attributable to the fact that the plasticization effect predominated significantly over the “thinning” effect. Nevertheless, for the PLA/SBS (95/5 w/w) blends, upon addition of increasing amounts (0, 1.0, and 3.0 wt %) of the S-MA compatibilizer, the *X*_c_ of the PLA matrices progressively decreased from 19.5% to 7.1%, disclosing that, as far as gradual compatibilization was concerned, the “thinning” effect prevailed over the plasticization effect; this phenomenon was also found in PP-*g*-maleic anhydride-compatibilized PP/polyamide-6 blends [35] and PLA/poly(3-hydroxybutyrate-*ran*-3-hydroxyhexanoate) blends compatibilized with a reactive epoxy resin [36]. From Table 1, similar observations were made for the PLA/SBS (90/10 w/w) blends in terms of their *X*_c_ changes with compositions (i.e., SBS and S-MA concentrations). Obviously, these two effects indicated the effective role of S-MA in the compatibilization of PLA/SBS (95/5 and 90/10 w/w) blends.

### 3.3. Effect of S-MA Compatibilization on the Thermal Decomposition Behavior of PLA/SBS Blends

**Figure 4:**
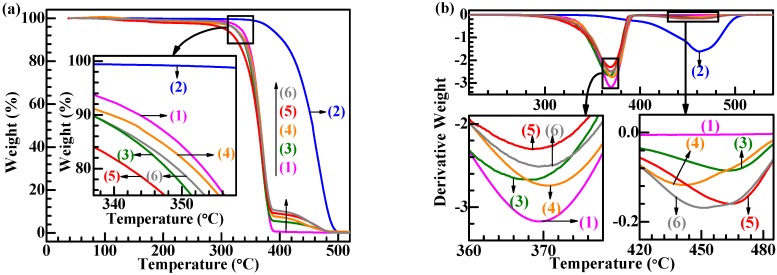
(**a**) Thermal gravimetric analysis (TGA) and (**b**) derivative thermogravimetric (DTG) thermograms at a heating rate of 10 °C/min for (1) (neat) PLA, (2) (neat) SBS triblock copolymer, blends of PLA and SBS (95/5 w/w) (3) uncompatibilized and (4) compatibilized with 3.0 wt % of S-MA, and blends of PLA and SBS (90/10 w/w) (5) uncompatibilized and (6) compatibilized with 3.0 wt % of S-MA. In graph (**a**), the inset is a magnification of the onset regions of thermal decomposition; in graph (**b**), the insets in the lower left and upper left corners, respectively, are magnifications of the two sequential maximum decomposition-rate regions.

To investigate the influence of S-MA compatibilizer inclusion on the thermal decomposition behavior of the PLA/SBS blends, TGA and derivative thermogravimetric (DTG) curves were analyzed, as illustrated in Figure 4a,b, respectively, for the (neat) PLA (Traces 1), the (neat) SBS (Traces 2), the different compositions, 95/5 (Traces 3 and 4) and 90/10 (Traces 5 and 6) (w/w), of PLA/SBS blends uncompatibilized (Traces 3 and 5) and compatibilized with 3.0 wt % of S-MA (Traces 4 and 6). The DTG curves shown in Figure 4b were analyzed and summarized in Table 2. As observed in Figure 4a,b, (neat) PLA and SBS exhibited a single-maximum-rate decomposition behavior, while the PLA/SBS blends displayed a double-maximum-rate decomposition behavior, where the weight losses at 300–400 and 400–500 °C, respectively, were primarily attributed to PLA matrix (cf. Traces 1) and SBS dispersed-phase (cf. Traces 2) decompositions.

From the magnified inset of Figure 4a, it is observed that the TGA traces (Traces 3 and 5) of the PLA/SBS blends fell below those (Traces 1 and 2) of (neat) PLA and SBS, suggesting that the thermal stability decreased upon blending of PLA and SBS, possibly due to the introduction of a lot of unstable interfaces that were susceptible to thermal energy. Upon S-MA compatibilization, the PLA/SBS blends displayed a higher thermal stability (Trace 4 above 3, and Trace 6 above 5); this revealed that the compatibilization was effective in that it led to stronger interfacial adhesion and thus reduced the “interfaces” effect to improve the interfacial thermal insusceptibility.

Further observed from the magnified insets of Figure 4b was a convergence of the two maximum decomposition-rate temperatures (*T*_md_’s) of the PLA/SBS blends upon S-MA compatibilization (Trace 4 vs 3, and Trace 6 vs 5), quantified in Table 2 by comparisons of the (*T*_md2_ − *T*_md1_) values (71.9 < 96.2 °C, and 79.0 < 96.2 °C). As revealed by the *T*_g_ convergence of a compatibilized polymer blend, this convergence of *T*_md_, although still to be addressed, might constitute another indicator of the enhanced interfacial compatibility of polymer blends, e.g., S-MA compatibilized the PLA/SBS blends investigated in this work.

### 3.4. Effects of S-MA Compatibilization on the Rheological Properties of PLA/SBS Blends

**Figure 5:**
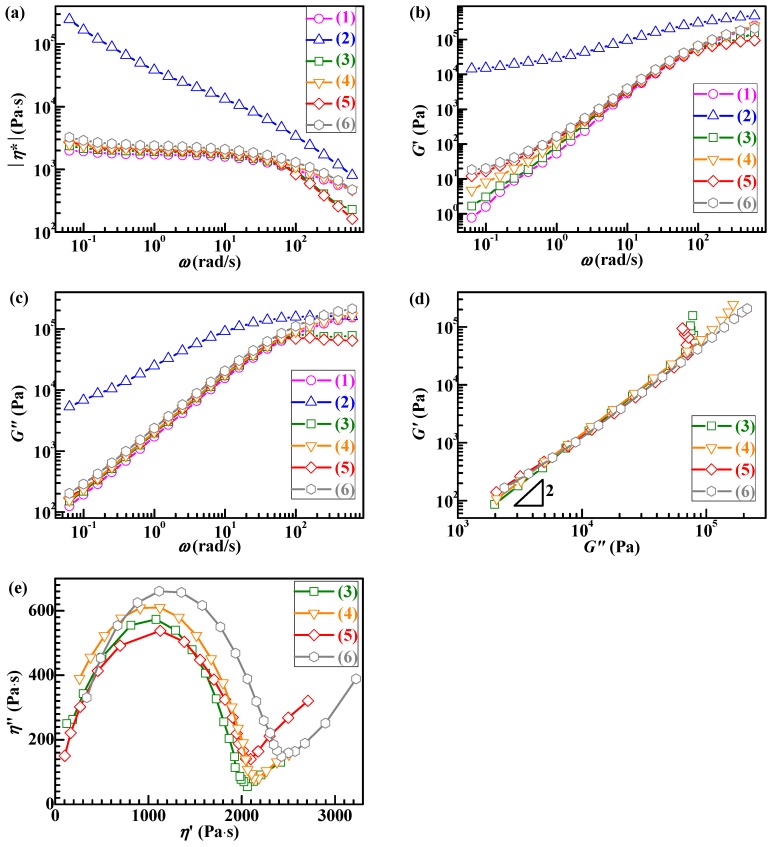
(**a**) Complex viscosity magnitude (|η^*^|), (**b**) dynamic storage modulus (*G*′), and (**c**) dynamic loss modulus (*G*″) as functions of angular frequency (*ω*), (i.e., log|η^*^| vs log*ω*, log*G*′ vs log*ω*, and log*G*″ vs log*ω*, respectively), (**d**) Han (i.e., log*G*′ vs log*G*″) plots, and (**e**) Cole–Cole (i.e., *η*″ vs *η*′) plots, which were obtained from the frequency-sweep tests at 180 °C in the oscillatory shear mode, for (1) (neat) PLA, (2) (neat) SBS, blends of PLA and SBS (95/5 w/w) (3) uncompatibilized and (4) compatibilized with 1.0 wt % of S-MA, and blends of PLA and SBS (90/10 w/w) (5) uncompatibilized and (6) compatibilized with 1.0 wt % of S-MA.

Figure 5 shows the plots of log|η^*^|, logarithm of complex viscosity magnitude, vs log*ω*, logarithm of angular frequency (Figure 5a), log*G*′, logarithm of dynamic storage modulus, vs log*ω* (Figure 5b), log*G*″, logarithm of dynamic loss modulus, vs log*ω* (Figure 5c), log*G*′ vs log*G*″ (i.e., Han plots) (Figure 5d), and *η*″ vs *η*′ (i.e., Cole–Cole plots) (Figure 5e) for (neat) PLA (Plots 1), (neat) SBS (Plots 2), different compositions, 95/5 (Plots 3 and 4) and 90/10 (Plots 5 and 6) (w/w), of PLA/SBS blends uncompatibilized (Plots 3 and 5) and compatibilized with 1.0 wt % of S-MA (Plots 4 and 6). It is seen from Figure 5a that, in the whole *ω* range investigated, (neat) SBS (Plot 2) exhibited a shear-thinning behavior, while the PLA matrix-based materials (Plots 1 and 3–6) displayed a Newtonian fluid behavior at low *ω* values but a shear-thinning behavior at higher *ω* values. Upon S-MA compatibilization, |η^*^| of the PLA/SBS blends increased across the *ω* investigated (Plot 4 above 3 and Plot 6 above 5 in Figure 5a), which indicated that S-MA did act as an effective compatibilizer for the PLA/SBS blends. Similar observations (Plot 4 above 3 and Plot 6 above 5) were made in Figure 5b and c, also suggestive of the effectiveness of the S-MA compatibilizer. 

In the Han plots (Figure 5d), the slopes in the low-frequency terminal region (lower left corner) were apparently all smaller than 2, revealing a two-phase (i.e., heterogeneous) morphology [37,38] of the PLA/SBS blends regardless of their compatibilization. However, in the higher frequency terminal region (upper right corner), the uncompatibilized blends (Plots 3 and 5) showed more significant, steeper ramifications than the compatibilized ones (Plots 4 and 6), which is hypothetically a consequence of the slippage [18,22] at higher shear rates at the uncompatibilized PLA–SBS interphase, which induced a weak melt viscosity effect. In other words, the interfacial adhesion of the PLA/SBS blends possibly became enhanced upon their compatibilization with S-MA, which led to much smaller ramifications of the compatibilized blends compared with the uncompatibilized ones. That is, S-MA compatibilization was probably successful.

The effect of S-MA inclusion on the compatibility of the PLA/SBS blends was also illustrated with the Cole–Cole plots (Figure 5e). According to references [39,40], a Cole–Cole plot having only a single semicircle without a following tail or second semicircle indicates a homogeneous morphology, that is, any semicircle with a tail or second semicircle suggests a multiphase (i.e., heterogeneous) morphology. It is observed from Figure 5e that all the Cole–Cole plots displayed a semicircle with a tail, revealing a two-phase morphology of the PLA/SBS blends, irrespective of their compatibilization. Nevertheless, it is seen that the semicircle radii of the S-MA-compatibilized PLA/SBS blends (Plots 4 and 6) were larger than those of the uncompatibilized blends (Plots 3 and 5) (Figure 5e). This was obviously due to the fact that there were better interfacial adhesion and hence longer chain segmental relaxation times in the compatibilized blends [41], which could further be attributed to increases in both thickness and chain entanglement degree in the interfacial layers. In this context, S-MA served as an effective compatibilizer for the PLA/SBS blends.

### 3.5. Effects of S-MA Compatibilization on the Mechanical Properties of PLA/SBS Blends

**Figure 6:**
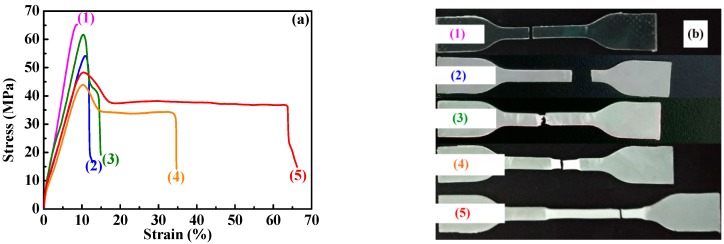
(**a**) Stress–strain behaviors and (**b**) images of typical fractured tensile specimens for (1) (neat) PLA, blends of PLA and SBS triblock copolymer (95/5 w/w) (2) uncompatibilized and (3) compatibilized with 1.0 wt % of S-MA, and blends of PLA and SBS (90/10 w/w) (4) uncompatibilized and (5) compatibilized with 1.0 wt % of S-MA.

Tensile and impact tests of the PLA/SBS blends were carried out to evaluate the compatibilization effect of the added S-MA. Figure 6a shows the typical tensile stress–strain curves of (neat) PLA (Curve 1) and of the different PLA/SBS blends, with compositions 95/5 (Curves 2 and 3) and 90/10 (Curves 4 and 5) (w/w), uncompatibilized (Curves 2 and 4) and compatibilized with 1.0 wt % of S-MA (Curves 3 and 5); images of the tensile fractured specimens are given in Figure 6b. It is seen in Figure 6a,b that, compared with a relatively brittle fracture behavior of (neat) PLA (1), the PLA/SBS blends (2–5) displayed pronounced yield behaviors with lower tensile strengths (*σ*_y_’s), larger elongations at break (*ε*_b_’s), and “necking” phenomena corresponding to strain softening behaviors, obviously due to the incorporation of small amounts (5–10 wt %) of the ductile SBS elastomer. More importantly, it is observed in Figure 6a,b that, compared with the uncompatibilized PLA/SBS blends (2 and 4), respectively, the compatibilized ones (3 and 5) exhibited higher *σ*_y_, larger *ε*_b_, and more stable “necking” phenomena; this contrast may originate from the considerably enhanced interfacial adhesion in the compatibilized blends, which infers that S-MA probably played an effective role in the compatibilization. It is noteworthy from Figure 6b that, upon effective compatibilization with S-MA, the dispersed-phase SBS might more efficiently stabilize the crazes [14,16,18] developed in the PLA matrix, resulting in more pronounced stress whitening, thus giving rise to PLA highly toughened by the added SBS.

**Figure 7:**
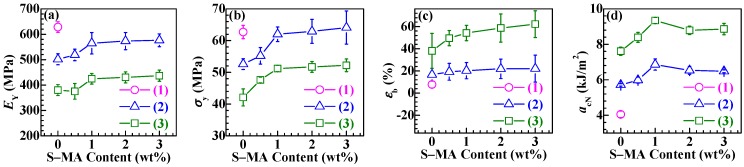
(**a**)–(**c**) Tensile properties, i.e., (**a**) Young’s moduli (*E*_Y_), (**b**) tensile strength (*σ*_y_), and (**c**) elongation at break (*ε*_b_), and (**d**) Charpy notched impact strength (*a*_cN_) for (1) (neat) PLA, (2) blends of PLA and SBS triblock copolymer (95/5 w/w) compatibilized with different amounts (0, 0.5, 1.0, 2.0, and 3.0 wt %) of S-MA), and (3) blends of PLA and SBS (90/10 w/w) compatibilized with different amounts of S-MA.

More quantitatively, the changes of the Young’s modulus (*E*_Y_), *σ*_y_, and *ε*_b_ (complete data are listed in Table S1) with S-MA concentration, which were analyzed from the stress–strain curves of the PLA/SBS blends compatibilized with various amounts (0, 0.5, 1.0, 2.0, and 3.0 wt %) of S-MA, were plotted and are shown in Figure 7a–c, respectively. It is seen that, with increases in the SBS fraction in the order of 0 wt % (i.e., neat PLA) (Points 1), 5 wt % (i.e., the 95/5 w/w blends) (Curves 2), and 10 wt % (i.e., the 90/10 w/w blends) (Curves 3), *E*_Y_ (Figure 7a) and *σ*_y_ (Figure 7b) of the blends decreased, while their *ε*_b_ (Figure 7c) increased, both monotonously. These observations, again, can be attributed to the increasing loading levels of the ductile SBS elastomer. Particularly worth noting from Figure 7a–c is that, for both compositions (95/5 and 90/10 w/w) of the PLA/SBS blends, *E*_Y_, *σ*_y_, and *ε*_b_ increased monotonically with steady increases in S-MA content from 0 to 1.0 wt %, beyond which they continued to increase insignificantly until 3.0 wt %, indicating that the 1.0 wt % concentration was adequate for the S-MA compatibilizer to maximally enhance the blends’ tensile properties. Again, this finding corroborates that S-MA indeed functioned as an effective compatibilizer to strengthen PLA–SBS interfacial adhesion, thereby minimizing the stress concentration effect at the interphase.

Most importantly, the Charpy notched impact strengths, *a*_cN_’s, of S-MA-compatibilized PLA/SBS blends were investigated against that of (neat) PLA, as shown in Figure 7d. It is observed in this figure that *a*_cN_ (Curves 2 and 3) of all the blends were noticeably higher than that (Point 1) of (neat) PLA, suggestive of the fact that the different levels of SBS incorporation, whether compatibilized with S-MA or not, did play a significant toughening role in the PLA matrix due to SBS ductile elastomeric nature. More interestingly, for each composition, 95/5 (Curve 2) or 90/10 (Curve 3) (w/w), of PLA/SBS blends, *a*_cN_ first improved and then deteriorated as the S-MA content increased monotonously from 0 to 3.0 wt %. In other words, there existed an intermediate (i.e., optimum) S-MA content at ~1.0 wt % that gave rise to a maximum in *a*_cN_, which was irrespective of the composition of the compatibilized blends. This observation, in accordance with that made above for the tensile properties, seemingly dictates that the 1.0 wt % of S-MA concentration constituted an optimized loading level for maximization of the mechanical (tensile and impact) properties of the PLA/SBS blends. The rationale behind the observation (i.e., a maximized *a*_cN_ at an intermediate S-MA content) may be presented as follows. As the S-MA compatibilizer content raised steadily, the increasingly enhanced compatibility of the PLA/SBS blends led to smaller SBS particles, along with better interfacial adhesion as well as thicker interphase layers (cf. Figure S1). According to the multiple crazing theory [42], positively, the smaller SBS particles contributed to a greater stabilization of the crazes developed in the PLA matrix to minimize their propagation into voids, cracks, etc. that caused impact failure, whereas, negatively, the smaller SBS particles also increased the difficulty of inducing the formation of new crazes that would favor impact energy dissipation. Consequently, *a*_cN_ maximization of the PLA/SBS blends occurred at a medium SBS particle size that corresponded to the intermediate S-MA compatibilizer content of ~1.0 wt %. Apparently, this explanation accords essentially with those in the literature [14,16,18]. Under these circumstances, Figure 7d impact testing results strongly confirmed that the incorporated S-MA was successful in the compatibilization of the PLA/SBS blends.

## 4. Conclusions

In an effort to improve the toughening efficiency of SBS-toughened PLAs, we synthesized, by surfactant-free emulsion copolymerization, a random copolymer, S-MA (S/MA = 75/25 mol/mol) and innovatively applied it as a third component to the compatibilization of PLA/SBS blends. To probe the compatibilization effect, the morphological, thermal transitional, thermal decompositional, rheological, tensile, and impact properties of melt-mixed PLA/SBS/S-MA blends were investigated and compared with those of melt-mixed PLA/SBS blends. SEM indicated that the impact-fractured surfaces of S-MA-compatibilized PLA/SBS blends displayed a more homogeneous, refined SBS dispersed-phase morphology than those of the uncompatibilized PLA/SBS blends. Upon S-MA compatibilization, the PLA/SBS blends exhibited a decreased relative degree of crystallinity, due, presumably, to a predominance of the so-called “thinning” effect, i.e., an increase in the volume fraction of the amorphous PLA–SBS interphase, as revealed by DSC. TGA showed that, compared with the uncompatibilized PLA/SBS blends, the compatibilized ones had higher thermal stability possibly as a result of the formation of more stable, thicker interfaces with stronger adhesion that were insusceptible to external thermal energy and presented a convergence of the maximum decomposition-rate temperatures of the PLA and SBS components, such as the *T*_g_ convergence predictably observed in DSC. Oscillatory shear rheometry suggested that the compatibilization of the PLA/SBS blends enhanced their |η^*^|, *G*′’, and *G*″’, reduced their ramification degrees in the high-frequency terminal region of the Han plots, and increased their semicircles radii in the Cole–Cole plots owing probably to the prolonged chain segmental relaxation times from increases in both thickness and chain entanglement degree in the interfacial layers. As the S-MA compatibilizer content increased monotonically from 0 to 3.0 wt %, *E*_Y_, *σ*_y_, and *ε*_b_, as well as the “necking” phenomenon following the strain softening behavior, of the PLA/SBS blends improves considerably and steadily until 1.0 wt %, beyond which they continue to increase but insignificantly, whereas the *a*_cN_ first improved and then deteriorated, showing an intermediate (i.e., optimum) S-MA concentration at ~1.0 wt % that gave rise to a maximum in *a*_cN_, hypothetically due to balanced effects of the medium-size SBS particles on the stabilization of preexisting crazes and the initiation of new crazes in the PLA matrix. In other words, the 1.0 wt % S-MA concentration practically constitutes an optimized loading level for maximization of the mechanical (tensile and impact) properties of the PLA/SBS blends. All these characterizations combined to evidence that S-MA acted effectively as a compatibilizer for the PLA/SBS blends. Block or graft copolymers have extensively been reported as typical compatibilizers [7,14,20], while, in this work, S-MA, a randomly sequenced copolymer synthesized for the first time in our laboratory, functioned as an efficient compatibilizer for SBS-toughened PLA blends. These results might help pave an alternative pathway to compatibilization of elastomer-toughened resin blends.

## Figures and Tables

**Table 1:** Thermal transitions, i.e., glass transition temperatures (*T*_g_’s), “cold”-crystallization (recrystallization) temperatures (*T*_cc_’s), “cold”-crystallization enthalpies (Δ*H*_cc_’s), melting temperatures (*T*_m_’s), and melting enthalpies (Δ*H*_m_’s), as well as relative degrees of (melt) crystallinity (*X*_c_’s), analyzed from Figure 3a DSC traces, for the (neat) PLA and the PLA/SBS (95/5 and 90/10 w/w) blends compatibilized with different concentrations (0, 1.0, and 3.0 wt %) of S-MA. Specifically, the *X*_c_ values were estimated from the Δ*H*_m_ values using Equation (1).

Composition	*T*_g_ (°C)	*T*_cc_ (°C)	Δ*H*_cc_ (J/g)	*T*_m_ (°C)	Δ*H*_m_ (J/g)	*X*_c_ (%)
PLA	61.6	137.2	1.1	165.7	1.3	1.4
PLA/SBS (95/5)	61.4	132.1	16.1	165.4	17.2	19.5
PLA/SBS/S-MA (95/5/1.0)	61.7	137.9	9.0	165.8	9.9	11.3
PLA/SBS/S-MA (95/5/3.0)	61.7	139.2	5.3	165.8	6.1	7.1
PLA/SBS (90/10)	61.7	139.5	5.1	166.3	6.0	7.2
PLA/SBS/S-MA (90/10/1.0)	61.7	140.1	4.0	166.6	4.8	5.8
PLA/SBS/S-MA (90/10/3.0)	62.0	140.2	2.5	166.8	2.8	3.4

**Table 2:** The temperatures (*T*_md1_ and/or *T*_md2_) at maximum decomposition rates, analyzed from Figure 4b DTG traces, for (neat) PLA, (neat) SBS, and PLA/SBS (95/5 and 90/10 w/w) blends compatibilized with different concentrations (0 and 3.0 wt %) of S-MA.

Composition	*T*_md1_ (°C)	*T*_md2_ (°C)	(*T*_md2_ − *T*_md1_) (°C)
(1) PLA	369.5	‒ ^1^	‒ ^1^
(2) SBS	‒ ^1^	459.5	‒ ^1^
(3) PLA/SBS (95/5)	367.6	463.8	96.2
(4) PLA/SBS/S-MA (95/5/3.0)	370.7	442.6	71.9
(5) PLA/SBS (90/10)	368.7	464.9	96.2
(6) PLA/SBS/S-MA (90/10/3.0)	370.3	449.3	79.0

^1^ Not applicable.

## Supplementary Materials

The following are available online at https://www.mdpi.com/2073-4360/11/5/846/s1, Figure S1: Scanning electron microscopy (SEM) micrographs of the impact-fractured surfaces of PLA/ SBS (90/10 w/w) blends (**a**) uncompatibilized, (**b**) compatibilized with 1.0 wt % of S-MA), and (**c**) compatibilized with 3.0 wt % of S-MA, Table S1: Young’s moduli (*E*_Y_), tensile strengths (*σ*_y_), elongations at break (*ε*_b_), and Charpy notched impact strengths (*a*_cN_) for (neat) PLA and blends of PLA and SBS (95/5 and 90/10 w/w) compatibilized with different concentrations (0, 0.5, 1.0, 2.0, and 3.0 wt %) of S-MA.
