# Supplementary material for: Effect of Poly(styrene-ran-methyl acrylate) Inclusion on the Compatibility of Polylactide/Polystyrene-b-Polybutadiene-b-Polystyrene Blends Characterized by Morphological, Thermal, Rheological, and Mechanical Measurements
Source: Polymers (Basel). 2019 May 10;11(5):846. doi: 10.3390/polym11050846 (PMC6572652; doi:10.3390/polym11050846)
Supplement: Supplementary file 1 [file polymers-11-00846-s001.pdf]

polymers

MDPI

Article

Effect of poly(styrene-*ran*-methyl acrylate) inclusion on the compatibility of polylactide/polystyrene-*b*-polybutadiene-*b*-polystyrene blends characterized by morphological, thermal, rheological, and mechanical measurements

Bocheng Wang [1], Zheng Tu [1], Chonggang Wu [1], Tao Hu [1], Xiaotao Wang [1], Shijun Long [1] and Xinghou Gong [1,*]

[1] Hubei Provincial Key Laboratory of Green Materials for Light Industry, Collaborative Innovation Center of Green Light-weight Materials and Processing, and School of Materials and Chemical Engineering, Hubei University of Technology, Wuhan, Hubei 430068, PR China; bochengwang@foxmail.com (B.W.); tuzheng666@foxmail.com (Z.T.); cgwu@mail.hbut.edu.cn (C.W.); hutao@mail.hbut.edu.cn (T.H.); xiaotaowang@aliyun.cn (X.W.); longshijun.hp@163.com (S.L.)

* Correspondence: gongxh@hbut.edu.cn (X.G.); Tel.: +86-1398-605-8907 (X.G.)

Received: xx April 2019; Accepted: xx April 2019; Published: xx April 2019

Supplementary Materials

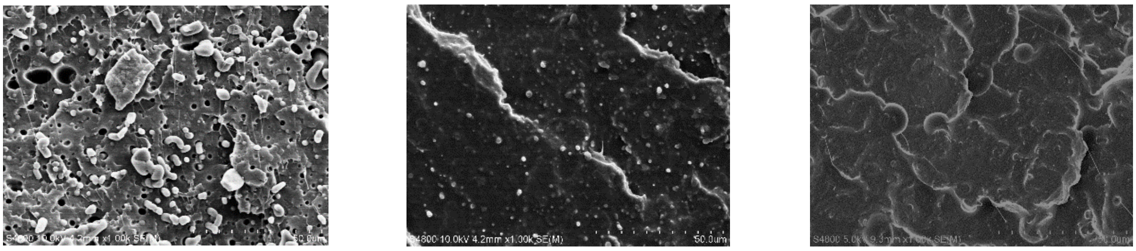

(a) (b) (c)

Figure S1. Scanning electron microscopy (SEM) micrographs of the impact fractured surfaces of polylactide (PLA)/polystyrene-*b*-polybutadiene-*b*-polystyrene (SBS) (90/10 w/w) blends (**a**) uncompatibilized, (**b**) compatibilized with 1.0 wt% of a poly(styrene-*ran*-methyl acrylate) (S–MA), and (**c**) compatibilized with 3.0 wt% of the S–MA.

Table S1. Young's moduli (E_Y's), tensile strengths (σ_y's), elongations at break (ε_b's), and Charpy notched impact strengths (a_{cN}'s) for a (neat) polactide (PLA) and blends of the PLA and a polystyrene-*b*-polybutadiene-*b*-polystyrene (SBS) (95/5 and 90/10 w/w) compatibilized with different concentrations (0, 0.5, 1.0, 2.0, and 3.0 wt%) of a poly(styrene-*ran*-methyl acrylate) (S–MA).

Composition	E_Y (MPa)	σ_y (MPa)	ε_b (%)	a_{cN} (kJ/m^2)
PLA	628.3 ± 21.1	62.7 ± 2.1	7.7 ± 3.1	4.1 ± 0.2
PLA/SBS (95/5)	501.4 ± 21.7	52.6 ± 1.7	16.6 ± 5.7	5.7 ± 0.1
PLA/SBS/S–MA (95/5/0.5)	518.6 ± 22.6	55.2 ± 2.6	19.2 ± 7.6	6.0 ± 0.2
PLA/SBS/S–MA (95/5/1.0)	564.7 ± 42.3	62.0 ± 2.3	20.2 ± 7.3	6.9 ± 0.3
PLA/SBS/S–MA (95/5/2.0)	573.2 ± 33.8	62.9 ± 3.8	21.9 ± 8.8	6.5 ± 0.2
PLA/SBS/S–MA (95/5/3.0)	576.3 ± 25.2	64.1 ± 5.2	22.1 ± 12.2	6.5 ± 0.1
PLA/SBS (90/10)	380.0 ± 21.7	42.1 ± 2.7	38.1 ± 15.7	7.6 ± 0.2

PLA/SBS/S–MA (90/10/0.5)	375.1 ± 30.9	47.6 ± 0.9	49.6 ± 6.9	8.4 ± 0.3
PLA/SBS/S–MA (90/10/1.0)	424.8 ± 21.0	51.2 ± 1.0	54.2 ± 7.0	9.3 ± 0.2
PLA/SBS/S–MA (90/10/2.0)	430.1 ± 21.7	51.7 ± 1.7	58.7 ± 12.7	8.8 ± 0.2
PLA/SBS/S–MA (90/10/3.0)	436.8 ± 22.0	52.2 ± 2.0	62.2 ± 12.0	8.9 ± 0.3
